# When does the placebo effect have an impact on network meta-analysis results?

**DOI:** 10.1136/bmjebm-2022-112197

**Published:** 2023-06-29

**Authors:** Adriani Nikolakopoulou, Anna Chaimani, Toshi A Furukawa, Theodoros Papakonstantinou, Gerta Rücker, Guido Schwarzer

**Affiliations:** 1 Institute of Medical Biometry and Statistics, Faculty of Medicine and Medical Center - University of Freiburg, Freiburg, Germany; 2 Centre of Research in Epidemiology and Statistics (CRESS-U1153), Inserm, Université Paris Cité, Paris, France; 3 Department of Health Promotion and Human Behavior, Kyoto University Graduate School of Medicine/School of Public Health, Kyoto, Japan

## Abstract

The placebo effect is the ‘effect of the simulation of treatment that occurs due to a participant’s belief or expectation that a treatment is effective’. Although the effect might be of little importance for some conditions, it can have a great role in others, mostly when the evaluated symptoms are subjective. Several characteristics that include informed consent, number of arms in a study, the occurrence of adverse events and quality of blinding may influence response to placebo and possibly bias the results of randomised controlled trials. Such a bias is inherited in systematic reviews of evidence and their quantitative components, pairwise meta-analysis (when two treatments are compared) and network meta-analysis (when more than two treatments are compared). In this paper, we aim to provide red flags as to when a placebo effect is likely to bias pairwise and network meta-analysis treatment effects. The classic paradigm has been that placebo-controlled randomised trials are focused on estimating the treatment effect. However, the magnitude of placebo effect itself may also in some instances be of interest and has also lately received attention. We use component network meta-analysis to estimate placebo effects. We apply these methods to a published network meta-analysis, examining the relative effectiveness of four psychotherapies and four control treatments for depression in 123 studies.

**WHAT IS ALREADY KNOWN ON THIS TOPIC**

- Placebo effect might mask true associations between treatments but it is not clear how this affects meta-analysis results.

**WHAT THIS STUDY ADDS**

- This study shows that factors that differentiate placebo effect within studies are likely to bias evidence synthesis treatment effects. We also show that under certain assumptions, when we can assume additivity of effects and equal placebo effect within and across studies, we can disentangle placebo from treatment effects.

**HOW THIS STUDY MIGHT AFFECT RESEARCH, PRACTICE OR POLICY**

- Assessing the robustness of meta-analysis results accounting for the role of placebo effect will provide valuable information to clinicians and patients.

## Introduction

Many definitions have been proposed for placebo, from ‘a medicine given more to please than to benefit’ in the Shorter Oxford Dictionary of 1811, to ‘something that is intended to act through a psychological mechanism’,^1^ to more recent definitions, for example, ‘the effect of the simulation of treatment that occurs due to a participant’s belief or expectation that a treatment is effective’^2^ and ‘beneficial effects that are attributable to the brain–mind responses to the context in which a treatment is delivered rather than to the specific actions of the drug’.^3^ Different definitions reflect the time period in which they were proposed but also the scientific field within which placebo is studied. Research studies in neuroscience, psychology and medicine are constantly being undertaken, trying to tackle the mechanisms of placebo and its practical implications.^4–7^ In epidemiology, increased interest in placebo partly arises from concerns that large placebo effects may mask true clinical effects and bias results.^2^ Such concerns have led to a wave of research, alternative study designs^8–13^ and statistical methods,^14–21^ focused on assessing and controlling placebo effects.

Evidence synthesis techniques have also contributed to understanding placebo effects. In 1955, Henry Beecher collected 15 studies examining different diseases and found that 35% of all 1082 patients were satisfactorily relieved by a placebo.^22^ In his research article ‘The powerful placebo’, using in principle an evidence synthesis perspective, Beecher recognised placebo as a clinically important factor, rendering the 35% an often-cited figure in favour of the argument that placebo can be an important medical treatment. Almost half a century later, Hróbjartsson and Gøtzsche questioned the significance of the placebo effect wondering ‘Is the placebo powerless?’ in a research article in which they performed a meta-analysis of 114 randomised trials and found little evidence that placebos have powerful clinical effects.^23^ Since then, a plethora of pairwise meta-analyses, meta-regression and network meta-analyses (NMA) have been conducted to investigate, among others, the debatable rise of placebo response rates^24–29^ and the influence of patient characteristics and several study-specific factors on placebo responses,^30 31^ such as the probability of receiving placebo^32–36^ and the type of placebo.^37–40^

The classic paradigm has been that placebo-controlled randomised trials are focused on estimating the treatment effect, that is, the relative effect of treatment compared with placebo. However, the magnitude of placebo effect itself may also in some instances be of interest and has also lately received attention.^2^ It is worth noting that placebo effects are not expected to be equally impactful across medical fields. Although Hróbjartsson and Gøtzsche concluded to an in general ‘powerless placebo’, they did find a significant effect between placebo and no treatment in studies with continuous subjective outcomes and in studies involving the treatment of pain.^23^ In this paper, we aim to shed light as to when the placebo effect is likely to bias pairwise and NMA treatment effects and propose instruments from the evidence synthesis methodological toolkit that can be used to estimate placebo effects.

### Definitions

**Figure 1:**
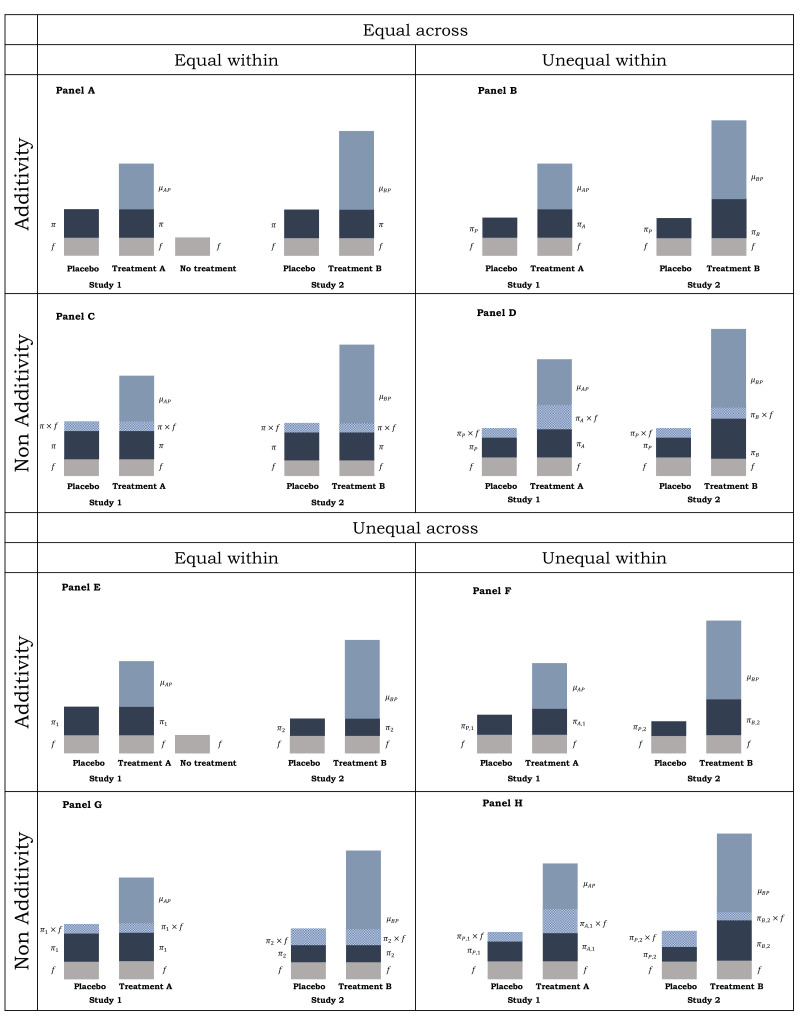
Schematic representation of placebo response and treatment response, decomposed to placebo effect, non-specific effects and treatment effect under different assumptions. π , placebo effect; f: , non-specific effects; $\mu_{AP}$ , relative treatment effect between A and P ; $\mu_{BP}$ , relative treatment effect between B and P .

Let us focus on figure 1 panel A Study 1 to introduce the definitions to be used throughout the paper. The three included treatments Placebo, Treatment A and No treatment are denoted as P , A and N , respectively. We define placebo response as the response that would be observed for each participant if assigned to placebo. Placebo response consists of both a possible placebo effect (π) as well as other possible non-specific effects (f) . These non-specific effects include the natural course of the disease or other mechanisms that lead to improvement such as the Hawthorne effect, the effect of responding to being observed and assessed.^2 41^ Treatment response, on the other hand, is defined as the response that would be observed for each participant if assigned to treatment (here treatment A ). It consists of three components: placebo effect, non-specific effects and true relative treatment effect between A and P (in the remainder to be called treatment effect and denoted as $\mu_{AP}$ ). Responses to treatments P , A and N for study i are denoted as $y_{A,i},y_{P,i}$ and $y_{N,i}$ respectively.

In a two-arm placebo-controlled trial, comparing treatment A with placebo, it is not possible to isolate the placebo effect from non-specific effects. Indeed, what is often investigated is the placebo response, which however is a combined effect that includes placebo effect and additional non-specific effects. To elucidate the placebo effect, one would need to subtract any non-specific effects from the observed placebo response. A no-treatment control arm serves this purpose (third arm in figure 1 panel A Study 1); the idea is that, due to randomisation, the non-specific effects will be the same across no-treatment control, placebo and active treatment and thus the placebo effect can be estimated by comparing the observed responses in the placebo arm and the no-treatment control arm.^2 42^

Miller and Rosenstein note that progress in understanding and estimating the placebo effect has been hampered by a lack of conceptual clarity, some of which has been due to confusion of the placebo effect with the placebo response.^15 42^ Notably, the apparent distinction between the conclusions of Beecher on one hand and Hróbjartsson and Gøtzsche on the other hand boils down to the definitions of placebo response and placebo effect.^22 23^ While Becher measured the placebo response, Hróbjartsson and Gøtzsche used studies with a no-treatment control arm to measure the placebo effect, isolating it from other non-specific effects.

### When should the placebo effect be of concern for evidence synthesis?

The example used in Definitions makes a number of assumptions. In this section, we elaborate on what is implicitly assumed about the placebo effect in meta-analysis and how departures from the assumptions impact on the unbiased estimation of direct and indirect treatment effects. Figure 1 serves as a guide of the scenarios one may encounter in practice in systematic reviews of interventions and table 1 gives the mathematical formulation of the respective models.

**Table 1:** Models for placebo response and treatment response, decomposed to placebo effect, non-specific effects and treatment effect under different assumptions. π : placebo effect; f :non-specific effects; μAP : treatment effect. Responses to treatments P , A and N for study i are denoted as $y_{A,i},y_{P,i}$ and $y_{N,i}$ respectively. The random errors $\epsilon_{A,i},\epsilon_{P,i}$ and $\epsilon_{N,i}$ are assumed to be distributed normally with expectation 0. Panels numeration refer to panels of figure 1.

	Equal across					
	Equal within			Unequal within		
Additivity	Panel A	Study 1	$y_{A,1}=\mu_{AP}+f+\pi +\varepsilon_{A,1}$ $y_{P,1}=f+\pi +\varepsilon_{P,1}$ $y_{N,1}=f+\varepsilon_{N,1}$	Panel B	Study 1	$y_{A,1}=\mu_{AP}+f+\pi_{A}+\varepsilon_{A,1}$ $y_{P,1}=f+\pi_{P}+\mu_{P,1}$
		Study 2	$y_{B,2}=\mu_{BP}+f+\pi +\varepsilon_{B,2}$ $y_{P,2}=f+\pi +\varepsilon_{P,2}$		Study 2	$y_{B,2}=\mu_{BP}+f+\pi_{B}+\varepsilon_{B,2}$ $y_{P,2}=f+\pi_{P}+\mu_{P,2}$
Non-additivity	Panel C	Study 1	$y_{A,1}=\mu_{AP}+f+\pi +(\pi \times f)+\varepsilon_{A,1}$ $y_{P,1}=f+\pi +(\pi \times f)+\varepsilon_{P,1}$	Panel D	Study 1	$y_{A,1}=\mu_{AP}+f+\pi_{A}+(\pi_{A}\times f)+\varepsilon_{A,1}$ $y_{P,1}=f+\pi_{P}+(\pi_{P}\times f)+\varepsilon_{P,1}$
		Study 2	$y_{B,2}=\mu_{BP}+f+\pi +(\pi \times f)+\varepsilon_{B,2}$ $y_{P,2}=f+\pi_{2}+(\pi_{2}\times f)+\varepsilon_{P,2}$		Study 2	$y_{B,2}=\mu_{BP}+f+\pi_{B}+(\pi_{B}\times f)+\varepsilon_{B,2}$ $y_{P,2}=f+\pi_{P}+(\pi_{P}\times f)+\varepsilon_{P,2}$
	Unequal across					
	Equal within			Unequal within		
Additivity	Panel E	Study 1	$y_{A,1}=\mu_{AP}+f+\pi_{1}+\varepsilon_{A,1}$ $y_{P,1}=f+\pi_{1}+\varepsilon_{P,1}$ $y_{N,1}=f+\varepsilon_{N,1}$	Panel F	Study 1	$y_{A,1}=\mu_{AP}+f+\pi_{A,1}+\varepsilon_{A,1}$ $y_{P,1}=f+\pi_{P,1}+\varepsilon_{P,1}$
		Study 2	$y_{B,2}=\mu_{BP}+f+\pi_{2}+\varepsilon_{B,2}$ $y_{P,2}=f+\pi_{2}+\varepsilon_{P,2}$		Study 2	$y_{B,2}=\mu_{BP}+f+\pi_{B,2}+\varepsilon_{B,2}$ $y_{P,2}=f+\pi_{B,2}+\varepsilon_{P,2}$
Non-additivity	Panel G	Study 1	$y_{A,1}=\mu_{AP}+f+\pi_{1}+(\pi_{1}\times f)+\varepsilon_{A,1}$ $y_{P,1}=f+\pi_{1}+(\pi_{1}\times f)+\varepsilon_{P,1}$	Panel H	Study 1	$y_{A,1}=\mu_{AP}+f+\pi_{A,1}+(\pi_{A,1}\times f)+\varepsilon_{A,1}$ $y_{P,1}=f+\pi_{P,1}+(\pi_{P,1}\times f)+\varepsilon_{P,1}$
		Study 2	$y_{B,2}=\mu_{BP}+f+\pi_{2}+(\pi_{2}\times f)+\varepsilon_{B,2}$ $y_{P,2}=f+\pi_{2}+(\pi_{2}\times f)+\varepsilon_{P,2}$		Study 2	$y_{B,2}=\mu_{BP}+f+\pi_{B,2}+(\pi_{B,2}\times f)+\varepsilon_{B,2}$ $y_{P,2}=f+\pi_{P,2}+(\pi_{P,2}\times f)+\varepsilon_{P,2}$

### Placebo effects equal across and within studies and additivity holds

The first assumption we make in the example used in Definitions (figure 1 panel A Study 1) is that non-specific effects are equal across all treatment arms. This will be assumed to be true in the remainder of this paper. Second, it was assumed that placebo effects are equal across treatment arms within a study. Third, it was assumed that additivity holds, meaning that, in expectation, the response that would be observed for a treatment is equal to the response that would be observed for placebo, plus the treatment effect. Equivalently, additivity means that the amounts of non-specific effects, placebo effect and treatment effect are independent and do not act synergistically or antagonistically. We differentiate between additivity assumption and the assumption of equal placebo effects within and/or across studies.

The model for figure 1 panel A Study 1 is then given in table 1. The difference between treatment response and placebo response $\left(y_{A,1}-y_{P,1}\right)$ provides an unbiased estimate of the treatment effect $\mu_{AP}$ , which is estimated from individual studies and pairwise meta-analyses.^43^ Having another study examining treatment B versus placebo (figure 1 panel A Study 2) leads to a fourth assumption, that placebo effects are equal across studies evaluating different treatments. In such a situation, it follows that estimates of both direct treatment effects $\mu_{AP}$ and $\mu_{BP}$ as well as indirect treatment effect $\mu_{AB}$ are unbiased.

### Placebo effects equal within studies, unequal across studies and additivity holds

In this situation, the placebo effect may differ across studies. For example, placebo effects may be bigger in a two-arm rather than a three-arm study (which would include placebo and two active treatments), as participants will know that it is more likely to receive an active treatment. Some studies have indeed found an association between treatment effect and number of treatment arms in the study (probability of receiving placebo).^32–36^ Other study-specific factors, such as informed consent,^44^ participant–staff contact^45^ and type of placebo,^46 47^ may also differentiate placebo effects across studies.

However, such a differentiation is taken into account in random-effects NMA and does not per se bias pairwise and NMA treatment effects.^43^ Consider, for example, figure 1 panel E, which illustrates one treatment A versus placebo and one treatment B versus placebo study. The indirect relative treatment effect between A and B would then be an unbiased estimate of the true relative effect $\mu_{AP}-\mu_{BP}$ as placebo effects $\pi_{1}$ and $\pi_{2}$ cancel out.

### Placebo effects unequal within and across studies and additivity holds

Not all study-specific characteristics impacting placebo effects would leave NMA treatment effects unbiased. Consider, for example, figure 1 panel F. Such a differentiation of placebo effects within and across studies might occur and bias the estimation of $\mu_{AP}$ and $\mu_{BP}$ . This can be the result of unmasking as patients may suspect that they are in the active treatment due to the occurrence of adverse events, altering their expectations and potentially biasing the estimation of the treatment effect.^48^ To mitigate this possibility, active controls that would cause the same adverse events as the treatments have been proposed, but have been deemed impractical in clinical trial settings.^2^ More generally, any compromises in blinding of participants and/or assessors could lead to unmasking and consequently differentiate placebo effects within a study.

The model for figure 1 panel F, given in table 1, allows for different placebo effects across and within studies and implies that the treatment effect for study *i* is overestimated if $\pi_{A,i}>\pi_{P,i}$ . Including biased study treatment effects in pairwise meta-analysis or NMA will lead to biased direct and indirect treatment effects. Depending on the weight such biased studies receive in the meta-analysis, the results may be invalid.

### Placebo effects unequal within studies, equal across studies and additivity holds

In figure 1 panel B, placebo effects are differentiated within studies but are equal across studies, meaning that $\pi_{P,1}=\pi_{P,2}=\pi_{P}$ for placebo. Similarly, placebo effects for other treatments are assumed to be equal across studies, $\pi_{A,i}=\pi_{A}$ for any study i including treatment A. The model for figure 1 panel B is a special case of that of figure 1 panel F (table 1). In particular, the indirect treatment effect $\left(y_{A,1}-y_{P,1})-(y_{B,2}-y_{P,2}\right)$ is biased by $\pi_{A}-\pi_{B}$ and thus such a situation would also produce biased pairwise meta-analysis and NMA results. The situation depicted in figure 1 panel B is not very realistic to occur in practice.

### Violation of additivity assumption

The assumption of additivity made in figure 1 panels A, B, E & F has been a point of controversy in the literature^2^ as it may be unrealistic in several instances. Violation of the additivity assumption could happen if, for example, the placebo effect interacts with non-specific effects. In such a case, placebo could act either synergistically or antagonistically, for example, with natural healing of the body. However, such a violation would not always bias treatment effects. If the interaction π×f is equal within and across studies (figure 1 panel C) or even unequal across but equal within studies (figure 1 panel G), similar arguments as before can be made to show that direct and indirect treatment effects would be unbiased. On the other hand, unequal interactions within studies (figure 1 panels D & H) would result in biased direct and indirect treatment effects, rendering pairwise meta-analysis and NMA inappropriate tools for estimation. As with figure 1 panels B and F, figure 1 panel D can be considered as a special case of figure 1 panel H.

### Estimating placebo effects

The inclusion of a ‘second, untreated’ (no-treatment) control arm was suggested by Ernst and Resch as a way of disentangling the placebo effect from non-specific effects in placebo controlled trials.^8^ Such a no-treatment control arm serves as a control for placebo in the same way that placebo serves as a control for the active treatment. A series of concerns have been expressed regarding the inclusion of a no-treatment control arm, such as the unavoidable compromises in blinding which may alter expectations of participants about the level of benefit they can anticipate. Other study designs have been suggested, trying to overcome such concerns, like assuring participants that they are on a ‘waiting list’ for receiving active treatment. Alternative study designs include withholding^5 49^ or manipulating^10 50^ the information that participants are getting about the chances of receiving treatment, rendering the estimation of placebo effects less prone to bias but also raising ethical concerns.^51 52^

Given that a no-treatment control arm is included in a network of interventions, component NMA (CNMA) can be used to estimate the incremental placebo effect π , on top of treatment effects. Such a use of CNMA highlights the role of evidence synthesis and its methodological instruments in investigating the placebo effect but is possible only under certain network structures and assumptions. For a description of CNMA, interested readers can refer to studies by Welton *et al*, Rücker *et al* and Tsokani *et al*.^53–55^

In situations like those in figure 1 panels A and E, CNMA can be used to estimate π . CNMA estimates for components can be interpreted as incremental treatment effects. Taking, for example, the OR as effect measure, for a component C, the component effect is an incremental OR (iOR) defined as the OR of treatments X+C versus X for any treatment X.^56 57^ If additivity does not hold, but placebo effects and interaction effects are assumed to be equal within studies (figure 1 panels C and G), π can still be estimated using CNMA with interactions. In all other scenarios (figure 1 panels B, D, F or H), CNMA (with or without interactions) is not an appropriate instrument for estimating treatment and placebo effects.

## Application

We illustrate the above using as an example a published NMA by Michopoulos *et al*, who examined whether different control conditions can produce different effect estimates in psychotherapy studies for depression.^39^ We re-analysed the network by Michopoulos *et al* with the aim to estimate placebo effects along with treatment effects. All analyses were performed in R using netmeta.^58^

### Evidence base

Michopoulos *et al* included 123 studies with 12 596 participants investigating response as the primary outcome, defined as 50% or greater reduction in depressive symptoms from baseline to the end of the study. The network consisted of eight treatment nodes: four active interventions (Cognitive Behavioural Therapy (CBT), behavioural activation (BA), problem solving therapy (PST) and third wave CBT (3W)) and four control nodes (Waiting List, Pill Placebo, Psychological Placebo and No Treatment). The authors found important differences between effect estimates and concluded that different control conditions should not be lumped into a single group.

### Assumptions and synthesis method

**Figure 2:**
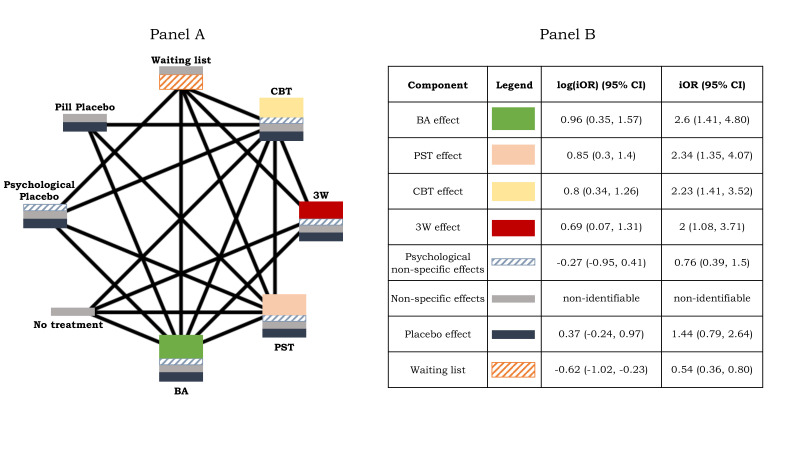
Panel A: Network plot of eight interventions for the treatment of depression, first analysed by Michopoulos *et al*.^39^ Intervention nodes consist of separate components, the heights of which are proportional to the respective log incremental ORs, estimated by component network meta-analysis. Height of non-specific effects is random as it is non-identifiable by component network meta-analysis. Panel B: Results of component network meta-analysis. BA, activation; CBT, cognitive behavioural therapy; iOR, incremental OR; PST, problem solving therapy; 3W, third wave CBT.

We hypothesise that ‘No Treatment’ represents non-specific effects while ‘Waiting List’ consists of non-specific effects plus an effect on its own, namely ‘waiting list effect’. ‘Pill Placebo’ consists of non-specific effects plus placebo effect and ‘Psychological Placebo’ includes on top psychological non-specific effects. These assumptions render the network to fall into the assumptions illustrated in figure 1 panel A and thus, CNMA can be used to quantify the effects of the components constituting each of the interventions. Figure 2 panel A illustrates the network of interventions along with the composition of each node. The height of each component is proportional to the respective log(iOR), estimated by CNMA.

## Results of CNMA

The results of CNMA are given in figure 2 panel B and table 2 shows the random-effects NMA treatment effects in a league table, estimated under the assumption that effects are additive and placebo effects are equal within and across studies (as illustrated in figure 1 panel A). The placebo effect is non-negligible resulting in an iOR of 1.44 with 95% CI 0.79 to 2.64, showing that the OR of a treatment versus any other is 1.44 times greater if placebo effect is included versus if it is not. For example, the OR of ‘Pill Placebo’ versus ‘No Treatment’ is equal to the iOR of placebo effect, 1.44 (95% CI 0.79 to 2.64). Non-specific effects are non-identifiable, as they are included in all treatment nodes in the network and thus cannot be isolated. Note that this follows from the assumption that non-specific effects are equal across all treatment arms, made throughout the manuscript. The iOR of ‘Waiting List’ and its 95% CI lie below 1, showing that NMA effects of any intervention against ‘Waiting List’ are bigger in comparison with NMA effects of the same intervention against any other control. This is also evident by the OR of ‘No Treatment’ versus ‘Waiting List’ which is estimated to be 0.54 with 95% CI 0.36 to 0.80 favouring ‘No Treatment’, equal to the iOR of ‘Waiting List’ (table 2). BA iOR is the largest, followed by PST effect, CBT effect and then 3W effect (figure 2 panel B). NMA ORs given in table 2 can also be derived by the iOR of figure 2 panel B. For example, the OR of CBT versus Pill Placebo is given as:

(2.23∗0.76∗iOR(non−specificeffects)∗1.44)/(iOR(non−specificeffects)∗1.44)=1.70

**Table 2:** Random-effects network meta-analysis (lower triangle) and pairwise meta-analysis (upper triangle) ORs with corresponding 95% CIs of the network by Michopoulos *et al*.^39^ For the lower triangle, ORs greater than 1 favour the treatment indicated in the column. For the upper triangle, ORs greater than 1 favour the treatment indicated in the row

CBT	4.83 (3.60 to 6.47)	1.87 (0.93 to 3.75)	2.38 (1.42 to 4.01)	2.17 (1.57 to 3.01)	0.86 (0.48 to 1.54)	1.14 (0.41 to 3.17)	1.04 (0.58 to 1.86)
4.59 (3.51 to 5.99)	WL	–	0.22 (0.02 to 2.14)	–	0.12 (0.04 to 0.33)	0.26 (0.10 to 0.73)	0.28 (0.15 to 0.51)
1.70 (0.99 to 2.93)	0.37 (0.20 to 0.67)	Pill placebo	–	–	0.45 (0.11 to 1.86)	0.62 (0.29 to 1.34)	–
2.23 (1.41 to 3.52)	0.49 (0.29 to 0.82)	1.31 (0.67 to 2.58)	Psychol placebo	–	0.07 (0.00 to 1.82)	0.55 (0.23 to 1.30)	–
2.46 (1.81 to 3.33)	0.54 (0.36 to 0.80)	1.44 (0.79 to 2.64)	1.10 (0.64 to 1.89)	No treatment	0.48 (0.16 to 1.43)	0.20 (0.09 to 0.47)	0.22 (0.05 to 0.92)
0.86 (0.56 to 1.32)	0.19 (0.12 to 0.30)	0.50 (0.26 to 0.96)	0.38 (0.21 to 0.71)	0.35 (0.21 to 0.57)	BA	1.51 (0.42 to 5.37)	1.14 (0.32 to 4.03)
0.95 (0.60 to 1.49)	0.21 (0.13 to 0.34)	0.56 (0.32 to 0.98)	0.43 (0.25 to 0.74)	0.39 (0.23 to 0.64)	1.11 (0.63 to 1.96)	PST	–
1.11 (0.73 to 1.70)	0.24 (0.16 to 0.38)	0.66 (0.33 to 1.29)	0.50 (0.27 to 0.93)	0.45 (0.27 to 0.75)	1.30 (0.74 to 2.29)	1.17 (0.64 to 2.14)	3W

BA, behavioural activation; CBT, cognitive behavioural therapy; PST, problem solving therapy; 3W, third wave CBT; WL, waiting list.

## Conclusions

In this paper, we showed how different assumptions about placebo effects impact on the validity of pairwise and NMA results. In summary, in situations depicted in figure 1 panels A and E, pairwise and NMA would produce unbiased estimates of treatment effects. When a no-treatment arm is included in the network, CNMA could also be employed to produce unbiased estimates of placebo effects. CNMA with interactions can be used for situations depicted in figure 1 panels C and G for the estimation of both treatment and placebo effects. For the rest of the cases, pairwise meta-analysis, NMA and CNMA results would not be valid and evidence synthesis should be precluded. In our example of psychotherapy studies in depression, we hypothesised that placebo effects are equal within and across studies. However, this might well not be true as in an open psychotherapy study, as it is typically the design of psychotherapy studies, explanations of treatments and subsequently expectations, might be different between active and control treatments or even between studies for the same control treatment. Thus, a situation as the one depicted in figure 1 panel F might be a more realistic assumption for the specific example. If data on blinding and adverse events are available, sensitivity analysis could also give hints on potential differentiations of placebo effect.

The placebo effect might also be interweaved with the tendency of patients to please the investigators by reporting improvements that have not occurred.^59^ In the original analysis by Michopoulos *et al* the funnel plot between active psychotherapies and various control conditions was highly asymmetric (online supplemental appendix F in^39^) showing that small studies were associated with larger treatment effects. A potential explanation is an association between small studies and compromised blinding of assessors which in turn could lead to bigger placebo effects in active treatments compared with control treatments in small studies. A further indication is the non-negligible meta-regression coefficient of 0.86 (95% CI −0.01 to 1.75) between blinding of assessor and NMA OR.

10.1136/bmjebm-2022-112197.supp1 Supplementary data

In line with such a possible mechanism, Holper and Hengartner argued that the rise in placebo effects could be explained by small study effects.^40^ Inclusion criteria and baseline risk, though, can also contribute to this phenomenon. As the debate over the rise of placebo^24–29^ has been mostly based on placebo responses, however, it would be interesting to investigate placebo effects over time using CNMA in networks of interventions that include a no-treatment control arm and a substantial number of studies to examine temporal trends.

It might also be of interest to investigate the impact of potential bias due to imbalance in placebo effects in NMA treatment effects. In order to do so, one can use influence analysis, originally developed to quantify the influence of a direct treatment effect to NMA treatment effects.^60^ Using this instrument, the relationship of the magnitude of $\pi_{A}-\pi_{P}$ and the NMA results can be shown. It is, however, restricted due to the fact that the imbalance of placebo effects in only one direct effect can be investigated. In the online supplemental appendix 1, we give an example of the potential use of influence analysis for examining the potential impact of imbalances between placebo effects. For a more thorough analysis, a simulation study, investigating several scenarios, where deviations from assumptions occurs, would be more informative.

In summary, factors that equally alter the placebo effect within a study might be of interest for estimating placebo effect, while factors that alter placebo effect within and across studies are important for properly estimating both placebo and treatment effects. By simultaneously investigating factors that may alter placebo effects across or within studies, NMA can shed light on their importance for producing unbiased estimates.

10.1136/bmjebm-2022-112197.supp2 Supplementary data

## Data Availability

Data are available upon reasonable request. Data and code are available upon reasonable request.
