# Supplementary material for: When does the placebo effect have an impact on network meta-analysis results?
Source: BMJ Evid Based Med. 2023 Jun 29;29(2):127–34. doi: 10.1136/bmjebm-2022-112197 (PMC10982636; doi:10.1136/bmjebm-2022-112197)
Supplement: Supplementary data [file bmjebm-2022-112197supp001.pdf]

Appendix

Use of influence analysis to investigate the impact of potential biased treatment effects

Let us consider a situation like the one depicted in Figure 1 Panel B Study 1. Then, the difference between placebo effects within a study, $\pi_A - \pi_P$, has an effect on the estimation of direct and indirect effects. Influence analysis (62) can be used to show the relationship between the magnitude of $\pi_A - \pi_P$ and the NMA results. In particular, influence analysis makes use of the projection matrix of a NMA model to quantify the influence of a direct treatment effect to NMA treatment effects. Let h_{AP}^{AB} denote the element of the projection matrix $\boldsymbol{H}$ containing the contribution of the direct effect A vs. P on the NMA treatment effect of A vs. B. Then, the influence function for a change of $\pi_A - \pi_P$ from 0 to δ is

$$IF_{AP,AB} = \delta \, h_{AP}^{AB}$$

Drawing such an influence function gives an indication how much NMA results (here NMA treatment effect A vs. B) will be if the direct comparison A vs. P changes by δ due, for example, to an anticipated imbalance of placebo effects within the comparison. Note, however, that such an analysis only takes the imbalance of placebo effects within one direct effect into account. In case that placebo imbalances are suspected in more than one direct comparison (as is the case in Figure 1 Panel B where placebo effects also differ in the treatment B vs. placebo comparison), such an investigation should be done separately for each of them.

Similar investigations can be performed when placebo effects are expected to differ both within and across studies, as in Figure 1 Panel F. Then, the study specific projection matrix will be used to create an influence function that would show the impact of $\pi_{A,i} - \pi_{P,i}$ to one or more NMA treatment effects.

Application of influence analysis on the Michopoulos et al. example

In the Michopoulos et. al. network of interventions, we investigate how much a difference of $\pi_{CBT} - \pi_{Pill\ Placebo} > 0$ would change the NMA odds ratios of three comparisons: CBT vs. Pill Placebo, CBT vs. PST and Waiting List vs. Pill Placebo. Such a

differentiation of placebo effects between CBT and Pill Placebo would divert our assumptions from Figure 1 panel A towards Figure 1 panel B. It could happen for example due to compromised blinding of assessors or when the two arms are provided with different explanations, leading to different credibility of treatment choices. Appendix Figure 1 shows how much of such a diversion would impact on erroneously inflating the NMA results. Among the three comparisons, the biggest influence would be pronounced in the comparison of CBT vs. Pill Placebo; from an estimated NMA odds ratio of 1.70 favoring CBT, a difference in the placebo effects of 1.10 would result to an NMA odds ratio of over 2.50.
