# Supplementary material for: When does the placebo effect have an impact on network meta-analysis results?
Source: BMJ Evid Based Med. 2023 Jun 29;29(2):127–34. doi: 10.1136/bmjebm-2022-112197 (PMC10982636; doi:10.1136/bmjebm-2022-112197)
Supplement: Supplementary data [file bmjebm-2022-112197supp002.pdf]

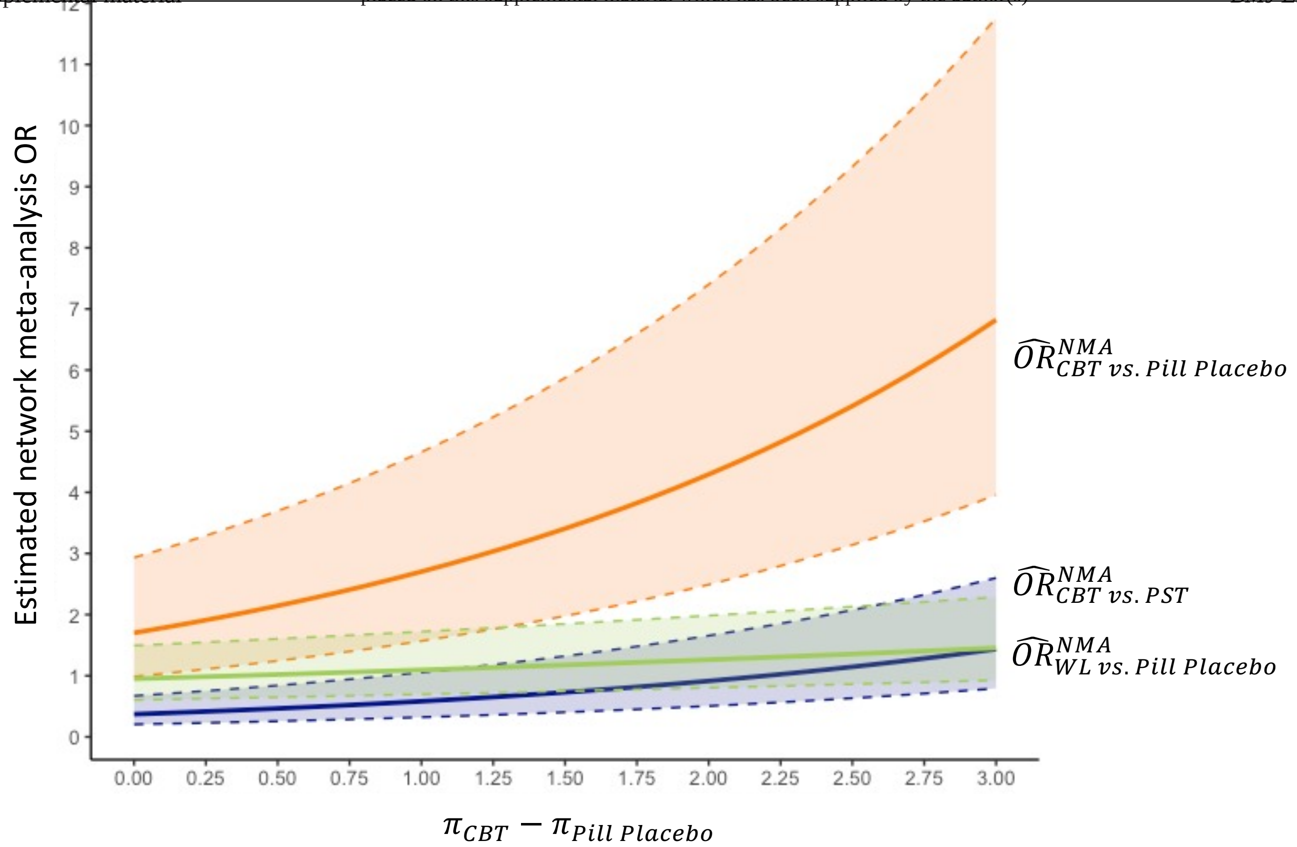
Estimated network meta-analysis OR
12
11
10
9
8
7
6
5
4
3
2
1
0
0.00
0.25
0.50
0.75
1.00
1.25
1.50
1.75
2.00
2.25
2.50
2.75
3.00
$\pi_{CBT} - \pi_{Pill\ Placebo}$
$\widehat{OR}^{NMA}_{CBT\ vs.\ Pill\ Placebo}$
$\widehat{OR}^{NMA}_{CBT\ vs.\ PST}$
$\widehat{OR}^{NMA}_{WL\ vs.\ Pill\ Placebo}$
